# Prediction of confirmed cases of and deaths caused by COVID-19 in Chile through time series techniques: A comparative study

**DOI:** 10.1371/journal.pone.0245414

**Published:** 2021-04-29

**Authors:** Claudia Barría-Sandoval, Guillermo Ferreira, Katherine Benz-Parra, Pablo López-Flores

**Affiliations:** 1 Nursing School, Universidad de las Américas, Concepción, Chile; 2 Faculty of Nursing, Universidad de Concepción, Concepción, Chile; 3 Department of Statistics, Universidad de Concepción, Concepción, Chile; 4 ANID - Millennium Science Initiative Program - Millennium Nucleus Center for the Discovery of Structures in Complex Data, Santiago, Chile; 5 Department of Primary Health Care, Servicio de Salud de Concepción, Concepcion, Chile; Universidad Nacional de Mar del Plata, ARGENTINA

## Abstract

**Background:**

Chile has become one of the countries most affected by COVID-19, a pandemic that has generated a large number of cases worldwide. If not detected and treated in time, COVID-19 can cause multi-organ failure and even death. Therefore, it is necessary to understand the behavior of the spread of COVID-19 as well as the projection of infections and deaths. This information is very relevant so that public health organizations can distribute financial resources efficiently and take appropriate containment measures. In this research, we compare different time series methodologies to predict the number of confirmed cases of and deaths from COVID-19 in Chile.

**Methods:**

The methodology used in this research consisted of modeling cases of both confirmed diagnoses and deaths from COVID-19 in Chile using Autoregressive Integrated Moving Average (ARIMA henceforth) models, Exponential Smoothing techniques, and Poisson models for time-dependent count data. Additionally, we evaluated the accuracy of the predictions using a training set and a test set.

**Results:**

The dataset used in this research indicated that the most appropriate model is the ARIMA time series model for predicting the number of confirmed COVID-19 cases, whereas for predicting the number of deaths from COVID-19 in Chile, the most suitable approach is the damped trend method.

**Conclusion:**

The ARIMA models are an alternative to modeling the behavior of the spread of COVID-19; however, depending on the characteristics of the dataset, other methodologies can better predict the behavior of these records, for example, the Holt-Winter method implemented with time-dependent count data.

## 1 Introduction

SARS-CoV-2, also called COVID-19, is an infectious contagious disease that originated as a pandemic of high impact on the health of the international population, causing death if not detected and treated on time. Regarding its origin, [1] pointed out that it is still being investigated and that the virus was initially declared to be of zoonotic origin due to its similarity to bat coronaviruses. However, other studies, such as [2, 3], have revealed the probable mutation of SARS-CoV (transmitted to humans through consumption of exotic animals) and MERS-CoV (transmitted from camels to humans) as the etiological source of COVID-19. Currently, prevention of the spread of COVID-19 has become the greatest concern worldwide, with governments and companies investing large amounts of money in public and private initiatives to ensure better control of the pandemic.

In emerging countries where the COVID-19 pandemic has deepened a social crisis with millions of people below the poverty line, millions of dollars have been lost in investments in different areas. The CEPAL report [4] discloses in an economic analysis carried out for Latin America and the Caribbean that the economy is projected to contract by 9.1%, which implies a ten-year fall to a level similar to that registered in 2010. Additionally, it indicates that this region will suffer an increase in the poverty rate of 37.3% and in the unemployment rate of 13.5%. Chile is no stranger to this reality; in the FLACSO-Chile Public Policy Program [5], it was mentioned that “economic activity would contract to 3.3% in 2020, accompanied by a significant decrease in trade flows, in the price of raw materials, especially the price of copper, expressed in an increase in unemployment and poverty figures, among many other economic and social effects”. In this scenario, it is imperative to study the behavior of the evolution of the number of cases with a confirmed diagnosis and the number of deaths from COVID-19 to ensure that government institutions can efficiently use available resources and generate timely public health policies.

Since the beginning of the pandemic, there has been great interest among researchers and research centers regarding trend analysis and prediction of the spread of COVID-19 in different cities worldwide. Regarding the process of modeling the COVID-19 pandemic, the literature indicates different techniques that address data recorded sequentially in time, suggesting theoretical support at both the inferential level of finite samples and the asymptotic property level for estimators of trends and temporal dependence of the data.

One of the most popular tools for analyzing and predicting data sequentially are time series models that allow predicting trends, breaks in structure, cycles, and unobserved values. For illustrative purposes, only a few representative contributions are listed below.

The Box-Jenkins methodology was used in [6] to model and make predictions of the spread of COVID-19 in Nigeria. In particular, the authors proposed a model of the type ARIMA of order 1,1,0 (ARIMA (1,1,0)) that provided a forecast for ten consecutive days of the virus. Additionally, [7] considered autoregressive time series models based on normal distributions of a mixture of two-piece scales, called TPSMNAR models, to model and predict cases with a confirmed diagnosis and recovered cases of COVID-19 worldwide. In the same context, [8] developed an ARIMA model to predict the epidemiological trend of COVID-19 prevalence and incidence utilizing Johns Hopkins data. [9] proposed disease trend models in Wuhan, Beijing, Shanghai, and Guangzhou using machine learning and mathematical model-based analysis with SEIR Models (susceptible, exposed, infectious, recovered) and neural network (NN) models. On the other hand, [10] proposed a comparative study of time series methods to estimate the percentage of active cases of COVID-19 since May 4, 2020, over the total population of ten countries. Among the methodologies proposed by these authors are the ARIMA model, Holt-Winters additive model (HWAAS), Exponential Smoothing with additive trend and additive seasonality, trigonometric seasonal formulation, Box-Cox transformation ARMA errors and component trend (TBAT), automatic forecasting procedure (Prophet), probabilistic forecasting with autoregressive recurrent networks (DeepAR) and neural basis expansion analysis for interpretable time series forecasting (N-BEATS). These authors used the root mean square error (RMSE) to assess the performance of each time series model and concluded that traditional statistical methods such as ARIMA and TBAT, in general, prevail over their deep learning counterparts, such as DeepAR and N-BEATS, and argue that the result is not a surprise given the lack of large amounts of data.

In the same line, [11] proposed a study to predict and model the number of COVID-19 cases using two methodologies: ARIMA and Exponential Smoothing methods. In this work, the authors mentioned that for different countries under study, there is no single model to describe the behavior of the number of cases, but according to the characteristics of the data, both methods are effective in describing the virus spread curves. Other methods have been studied, for example, [12] proposed a regression Poisson autoregressive model to understand contagion dynamics of COVID-19, [13] fitted the reported serial interval (mean and standard deviation (SD)) with a Gamma distribution and applied the “earlyR” package in R ([14]) to estimate *R*0 in the initial stage of the COVID-19 outbreak. Finally, [15] indicated that predictions of the COVID-19 pandemic using more complex models, such as the SEIR model, may not be more reliable than the use of a simpler SIR model. The reader is referred to the following authors and their references [16–18] to complement the review on other models used in COVID-19 predictions.

The main contribution of this study is the assessment of the practical usefulness of a wide range of possible statistical techniques that can be applied to the problem of estimating the severity of the pandemic, in terms of the total number of cases with a confirmed COVID-19 diagnosis and the total number of deaths from COVID-19 and to predict the time course of the pandemic. In particular, this study considers a comparative analysis between the ARIMA models, Exponential Smoothing, State Space models, the Bayesian approach and the GLARMA model.

The last three methods are utilize a Poisson distribution for count data with a local linear trend model. The estimation methods presented in this study will be useful to researchers who wish to investigate the spread characteristics of the COVID-19 pandemic, and such information may help governments or public health agencies make timely and informed decisions.

This study is organized as follows. In Section 2, a brief description of the dataset and the time series models used in this study are described in detail. In Section 3, the performance of the different models is examined by means of a statistical analysis that includes estimations of the parameters and goodness of fit of the residuals of each model. The main conclusions are summarized in Section 3.1.

## 2 Materials and methods

### 2.1 Dataset

**Fig 1:**
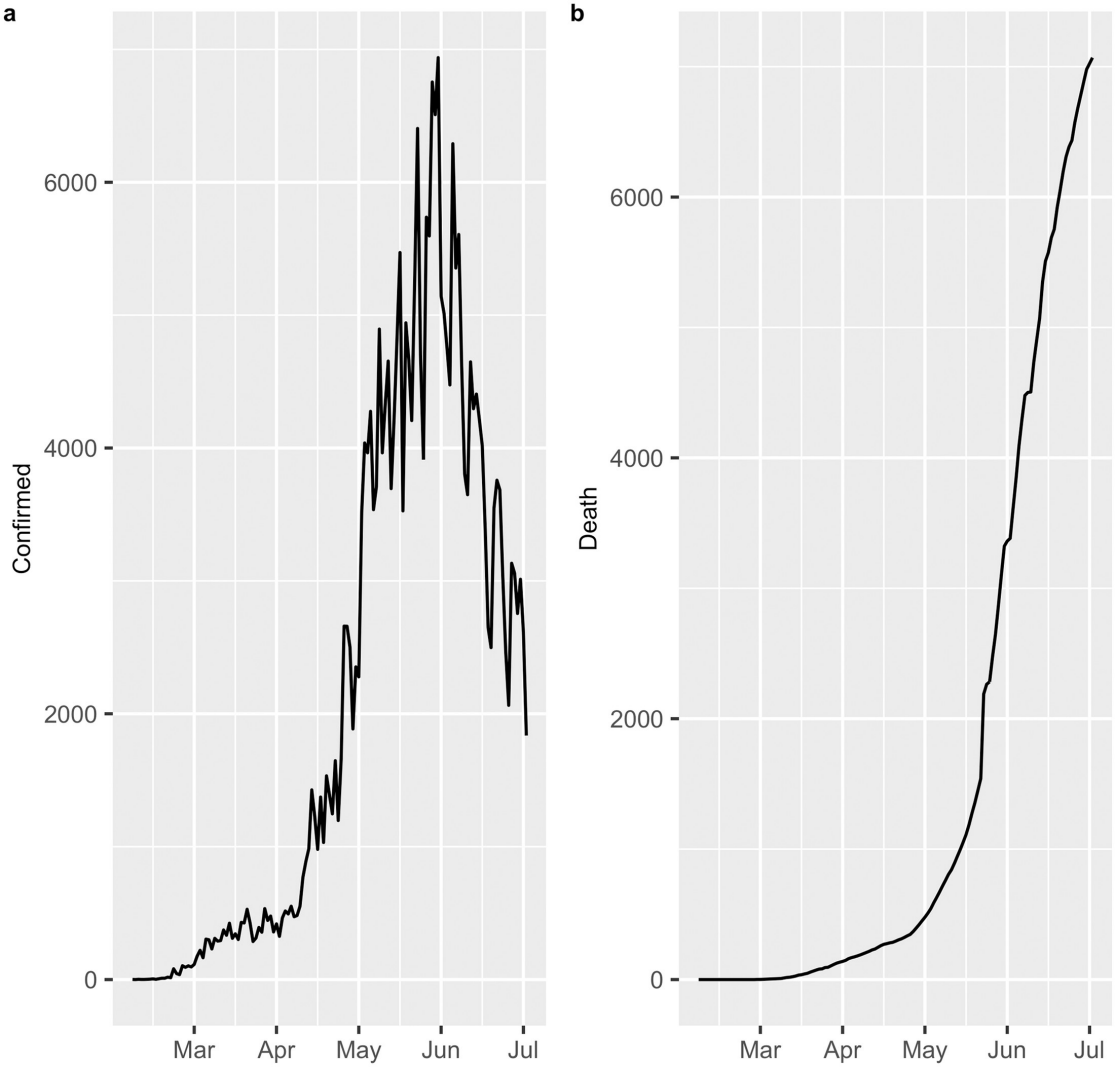
COVID-19 in Chile. (a) Confirmed cases. (b) Deaths.

In this study, an analysis was carried out to predict the number of confirmed COVID-19 diagnoses and deaths from COVID-19 in Chile, from March 2, 2020 to July 14, 2020. The data were obtained from the website of the Ministry of Science and Technology, Knowledge and Innovation http://www.minciencia.gob.cl/covid19. Fig 1(a) displays the number of cases with a confirmed diagnosis of COVID-19 in Chile, where a peak of infections is observed on June 15 that subsequently decreases until July 14. Furthermore, Fig 1(b) shows the deaths from COVID-19, where an exponential growth is observed up to the date of this study.

### 2.2 Model development

Time series models are an effective tool for modeling data recorded sequentially over time. The objective of this methodology is to capture the temporal dependence between observations through a mathematical model that allows the description of the main characteristics of the data. In general, these records present trends and seasonal components that can be modeled by different statistical techniques. In what follows, a description of the most widely used models in time series analysis will be presented, such as ARIMA(*p*, *d*, *q*) processes and a random walk with trend for count data, among others.

- ARIMA Model
  $$\begin{array}{c} {\left(1-B\right)}^{d}\phi \left(B\right)\;X_{t}=\mu +\theta \left(B\right)\;+\varepsilon_{t},\;\;\text{with}\;\;\left\{\varepsilon_{t}\right\}∼WN\left(0,\;\sigma^{2}\right),\;\;\text{Model}\;\text{1} \end{array}$$
  where *d* is the positive integer parameter of integration, *B* denotes the backward shift operator, *ϕ*(*z*) = 1 − *ϕ*_1_ *z* − ⋯ − *ϕ*_*p*_ *z*^*p*^ the autoregressive polynomial, *θ*(*z*) = 1 + *θ*_1_ *z* + ⋯ + *θ*_*p*_ *z*^*q*^ the moving average polynomial and {*ε*_*t*_} is a sequence of uncorrelated random variables with zero mean and variance *σ*^2^. Such a sequence is referred to as white noise, denoted by WN(0, *σ*^2^). [6] used this model to predict the propagation of COVID-19 in Nigeria; for more details of this model, the reader can review the time series book written by [19].
- Poisson Model
  Another model for working with time series count data is the Poisson process with a local linear trend model defined by
  $$\begin{array}{ccc} X_{t} & & ∼Po(e^{W_{t}}),\\ W_{t} & = & \mu_{t}+\varepsilon_{t},\;\;\text{with}\;\;\left\{\varepsilon_{t}\right\}∼N\left(0,\;\sigma_{\varepsilon }^{2}\right),\\ \mu_{t+1} & = & \mu_{t}+\nu_{t}+\xi_{t}\;\;\text{with}\;\;\left\{\xi_{t}\right\}∼N\left(0,\;\sigma_{\xi }^{2}\right),\;\;\;\;\;\;\text{Model}\;\text{2}\\ \nu_{t+1} & = & \nu_{t}+\zeta_{t},\;\;\text{with}\;\;\left\{\zeta_{t}\right\}∼N\left(0,\;\sigma_{\zeta }^{2}\right), \end{array}$$
  where *μ*_*t*_ is a random walk with a drift component given by *ν*_*t*_, and {*ε*_*t*_} is a Gaussian process of zero mean and variance $\sigma_{\varepsilon }^{2}$ that captures the extra variations of the time series. The errors {*ξ*_*t*_} and {*ζ*_*t*_} have similar distributional characteristics. Model 2 estimates and predictions will be made through two methodologies that are widely discussed in the literature ([20, 21], among other authors), namely,
  a) State Space models
  b) Bayesian analysis.
  For Model 2b), we consider a random walk of order 1, i.e.,
  $$\begin{array}{ccc} W_{t} & = & \alpha +\mu_{t}\\ \mu_{t} & = & \mu_{t-1}+\xi_{t}\;\;\text{with}\;\;\left\{\xi_{t}\right\}∼N\left(0,\sigma_{\xi }^{2}\right), \end{array}$$
  where *α* is the drift parameter, and $\sigma_{\xi }^{2}$ is the variance of the Gaussian process {*ξ*_*t*_}, which is estimated as precision $1/\sigma_{\xi }^{2}$ in the Bayesian framework.
- GLARMA(*p*, *q*) Model
  On the other hand, [22] provided another methodology for count data with a serial dependence in regression modeling of time series called generalized linear autoregressive moving average (GLARMA(*p*, *q*)) models defined by
  $$\begin{array}{ccc} X_{t} & & ∼Po(e^{W_{t}}),\\ W_{t} & = & \alpha +\beta t+Z_{t},\\ Z_{t} & = & \sum_{j=1}^{p}\phi_{j}\left(Z_{t-j}+\varepsilon_{t-j}\right)+\sum_{j=1}^{q}\theta_{j}\varepsilon_{t-j},\;\;\;\text{Model}\;\text{3} \end{array}$$
  where *β* is the intercept parameter of the deterministic trend *βt*, and {*ε*_*t*_} is defined as $\varepsilon_{t}=\frac{X_{t}-\mu_{t}}{\nu_{t}}$, with $\mu_{t}=e^{W_{t}}$ and *ν*_*t*_ is some scaling sequence.
- Holt’s Local Trend and Damped Trend Method
  Finally, [23, 24] proposed a methodology known as the Holt-Winters method. This method is a more general class than the Exponential Smoothing method which explains the level and trend of the data as follows
  $$\begin{array}{ccc} {\hat{X}}_{t+h|t} & = & \mu_{t}+hT_{t},\\ \mu_{t} & = & \alpha X_{t}+\left(1-\alpha \right)\left(\mu_{t-1}+T_{t-1}\right),\;\;\;\;\text{Model}\;\text{4a}\\ T_{t} & = & \beta \left(\mu_{t}-\mu_{t-1}\right)+\left(1-\beta \right)T_{t-1}, \end{array}$$
  where 0 ≤ *α* ≤ 1 is the smoothing parameter, and 0 ≤ *β* ≤ 1 is the smoothing parameter for the trend. The *h*-step ahead forecasts ${\hat{X}}_{t+h|t}$ are calculated using the smoothing equations for the level *μ*_*t*_ and the trend *T*_*t*_. [25] developed an Exponential Smoothing model designed to damp erratic trends defined as follows
  $$\begin{array}{ccc} {\hat{X}}_{t+h|t} & = & \mu_{t}+\left(\phi_{1}+\phi_{1}^{2}+\ldots +\phi_{1}^{h-1}\right)T_{t},\\ \mu_{t} & = & \alpha X_{t}+\left(1-\alpha \right)\left(\mu_{t-1}+\phi_{1}T_{t-1}\right),\;\;\;\text{Model}\;\text{4b}\\ T_{t} & = & \beta \left(\mu_{t}-\mu_{t-1}\right)+\left(1-\beta \right)\phi_{1}T_{t-1}, \end{array}$$
  where 0 < *ϕ*_1_ < 1 is damping parameter. Its dampens the trend to be more conservative for longer forecast horizons.

## 3 Results and discussion

In this section, we will analyze the performance of Models 1-4 described in Section 2.2. The data consists of the number of cases with a confirmed COVID-19 diagnosis and the number of deaths from COVID-19 in Chile from March 2 to July 14, 2020. In what follows, we describe the types of models used for the data as well as the commands and packages used from the free R software for the parameter estimates of the model. First, for Model 1, an ARIMA(0, 1, 2) model is applied with a drift to cases with a confirmed diagnosis of COVID-19 and an ARIMA(0, 2, 3) model without drift to deaths. Such estimates are obtained by using the Arima command from the forecast package. Model 2a) has been estimated using an exponential family of observations for State Space models, in particular we have implemented the KFAS package ([26]) to obtain the estimates of this model. In KFAS, this model can be written in a state space form by defining

model2a<-SSModel(Confirmed ~ SSMtrend(2, Q = list(NA,0)) + SSMcustom(Z = 1, T = 0, Q = NA, P1 = NA), distribution=“poisson”).

On the other hand, Model 2b) requires the Bayesian methodology to make inference on the parameters. One of the most widely used methods to develop Bayesian inference is the integrated nested Laplace approximation (INLA) approach, which is implemented in R through the package INLA; see [27] for more details. Here, the estimates are represented by the mean and SD of the posterior distribution for our models (e.g., the distribution of the parameters given the data). We assign noninformative priors for the model parameters. For the mean parameter *α*, a Gaussian distribution is proposed, i.e., *N*(0, 1/*τ*), *τ* = 0, and for the precision of the random walk *μ*_*t*_, a Gamma distribution was used, i.e., $1/\sigma_{\xi }^{2}∼Gamma\left(1,5\times 10^{-5}\right)$. For a neater and self-contained exposition, Table 1 shows the estimates of this model, that is, the mean and SD of the posterior distributions of the parameters as well as the credibility intervals with limits on the quantiles 0.025 and 0.975 respectively. Note that according to this table, all the parameters are significant at a 5% confidence level. In the case of Model 3, the estimates are based on the GLARMA package [28]. We proposed a GLARMA(2, 0) model for the data of cases with a confirmed diagnosis of COVID-19 and a GLARMA(1, 0) model for the data of deaths from COVID-19, where *ν*_*t*_ = 1 for both models. Finally, for the estimates of Model 4a and 4b, we used the holt command from the fore-cast package by adding the argument damped = TRUE.

**Table 1:** Posterior mean, standard deviation and 95% credible interval for the parameters under Model 2.

Dataset	Model	Parameter	Mean	SD	0.025 quant.	0.975 quant.
**Confirmed**	Model 2b	*α*	6.5670	0.016	6.534	6.597
		$\sigma_{\xi }^{2}$	0.0452	0.006	0.033	0.058
**Deaths**	Model 2b	*α*	5.104	0.045	5.009	5.188
		$\sigma_{\xi }^{2}$	0.185	0.030	0.132	0.248

Table 2 shows the estimates of the parameter and the estimated SD (in parentheses) of Models 1 and 3-4 for both time series (COVID-19 confirmed diagnosis and deaths). To test the significance of parameter estimates, we apply the *t*−statistic to Models 1-2a and 3 for both datasets. From this table, it can be seen that the *t*−statistics are highly significant at the 5% confidence level, while the sample mean *μ* of Model 1 is not statistically significant.

**Table 2:** Data of confirmed cases and deaths: Estimated parameters of Models 1, 2a, 3, and 4 on the COVID-19 series.

Dataset / Model	Parameter										
	*μ*	*ϕ*_1_	*ϕ*_2_	*θ*_1_	*θ*_2_	*θ*_3_	$\sigma_{\varepsilon }^{2}$	$\sigma_{\xi }^{2}$	$\sigma_{\zeta }^{2}$	*α*	*β*
**Confirmed** Model 1	17.8413 (22.5197)			−0.3405 (0.0843)	−0.1724 (0.0751)		286518				
Model 2a							0.9732	7.5697 (0.0825)	0.0549 (0.0191)		
Model 3		0.0172 (6.69*e*^−5^)	0.0109 (6.82*e*^−5^)							5.0590 (0.0130)	0.0290 (0.0001)
Model 4a							545.4529			0.3733	0.0348
Model 4b		0.9590					543.9325			0.3426	0.0341
**Deaths** Model 1				−0.8469 (0.0875)	−0.2824 (0.1012)	0.3321 (0.0949)	3999				
Model 2a							0.9732	8.8650 (0.0117)	0.0769 (0.0072)		
Model 3		0.0147 (5.5*e*^−5^)								2.2789 (0.0189)	0.05198 (0.0002)
Model 4a							65.4792			0.9999	0.1646
Model 4b		0.9800					65.9388			0.9999	0.1813

**Fig 2:**
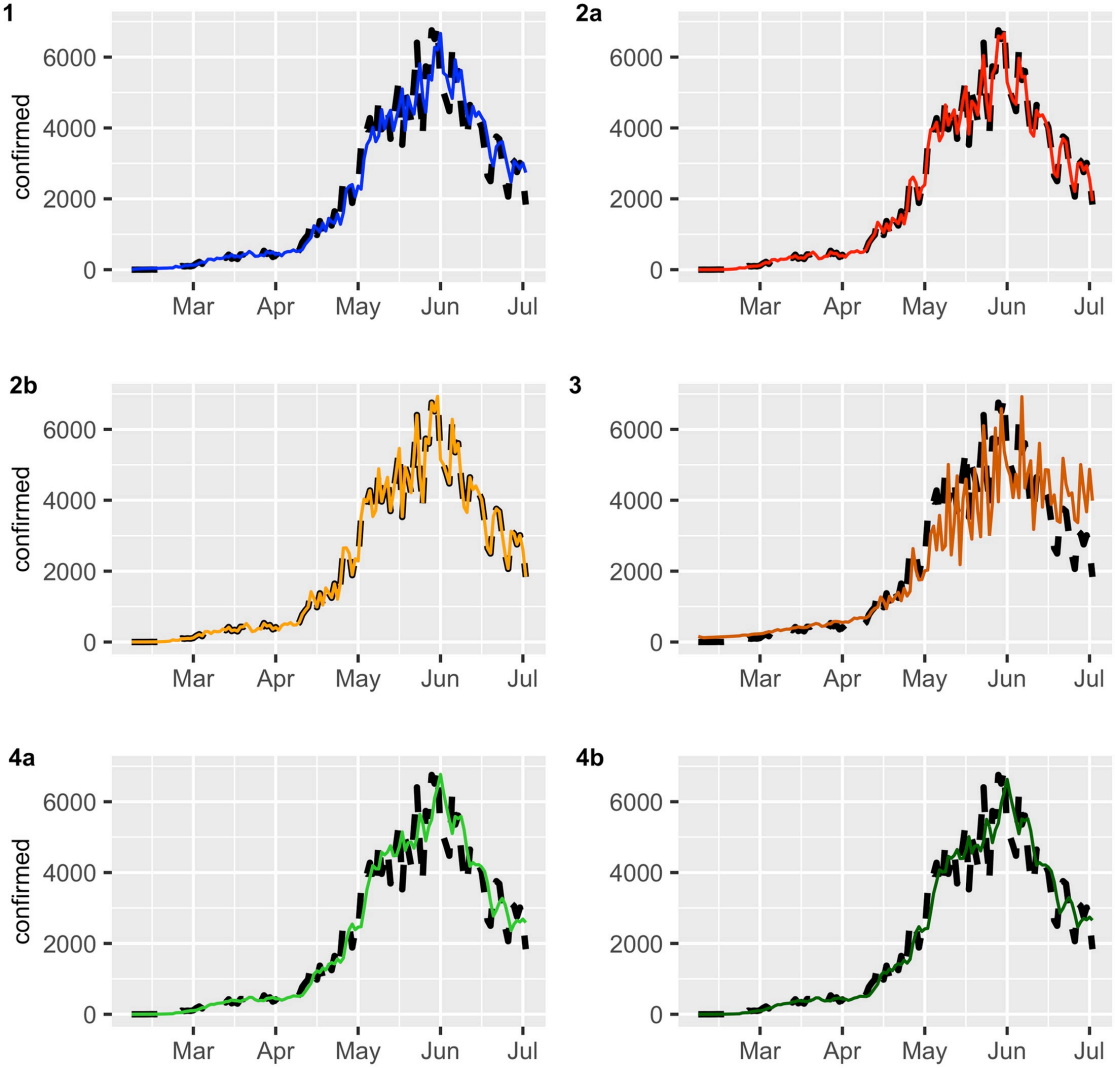
Confirmed cases (black dashed lines) versus fitted values (continuous line).

**Fig 3:**
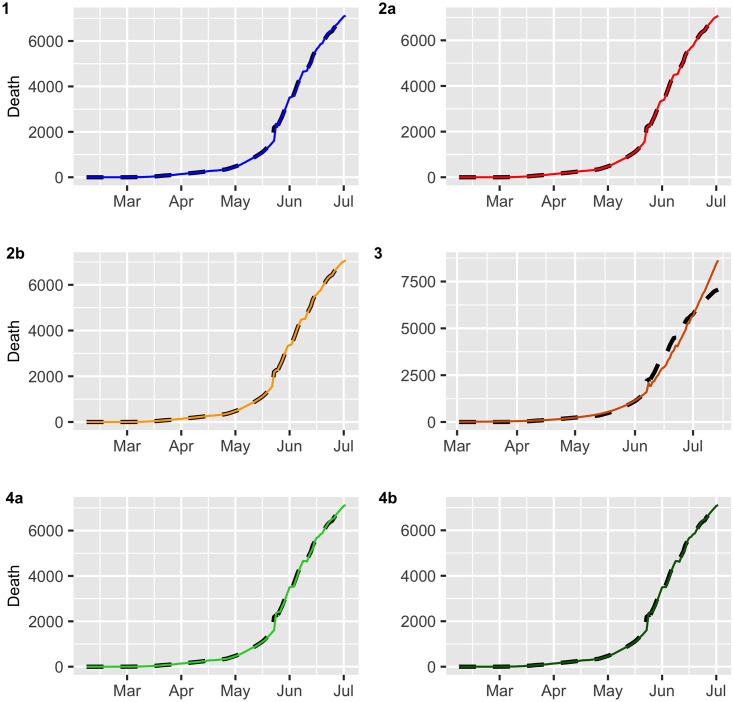
Deaths (black dashed lines) versus fitted values (continuous line).

**Fig 4:**
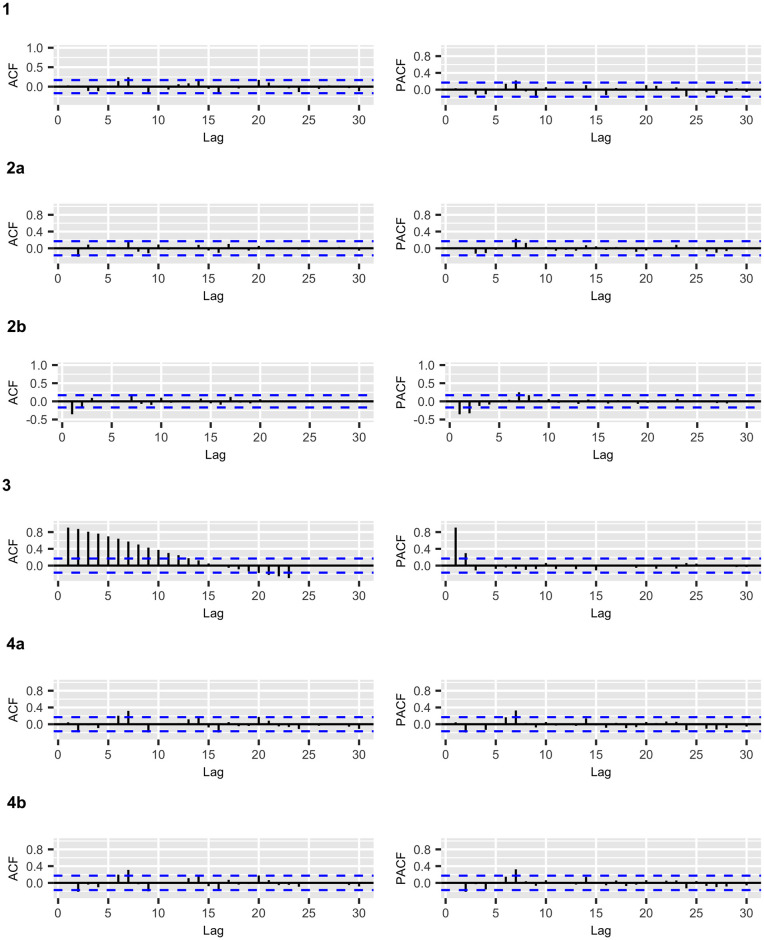
COVID-19 confirmed cases: ACF and PACF plots of the residuals for the fitted models.

**Fig 5:**
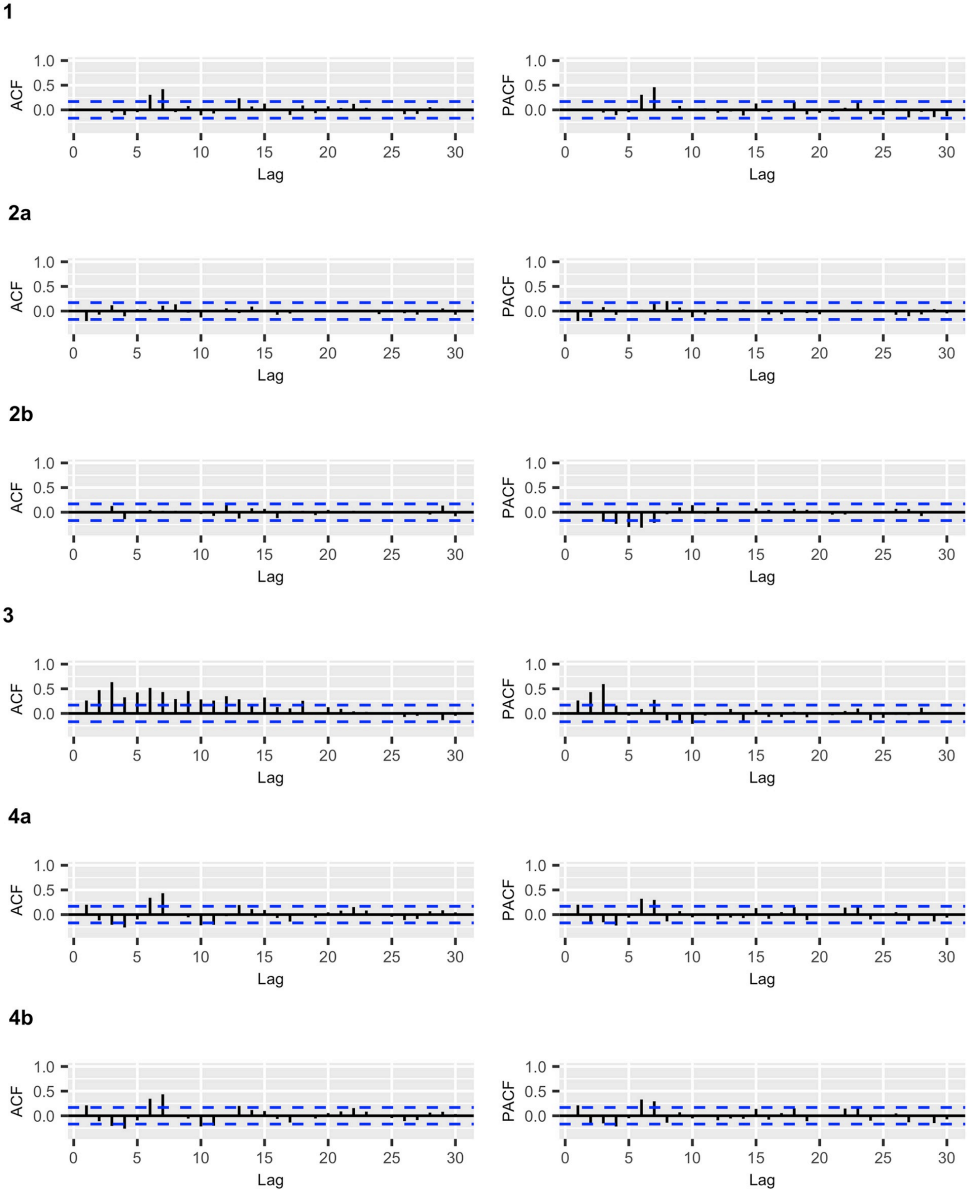
COVID-19 deaths: ACF and PACF plots of the residuals for the fitted models.

Figs 2 and 3 display the fitted values for each model, and the dashed lines represent the actual data, while the continuous line represents the fitted values that are shown in colored curves for Models 1-4. From these figures, we can conclude that the best model that captures the trend and temporal dependence structure is Model 2. Figs 4 and 5 present the sample autocorrelation function (ACF) and partial autocorrelation function (PACF) of the residuals of the estimated models for both datasets. From these figures, we can see that there are no significant autocorrelations in the residuals, except for Model 3. This result suggests that Model 3 is not adequate enough to capture the temporal dependence of the data under study. In what follows, we will evaluate the predictive power of the proposed models.

### 3.1 Analysis of ex post forecast accuracy

**Fig 6:**
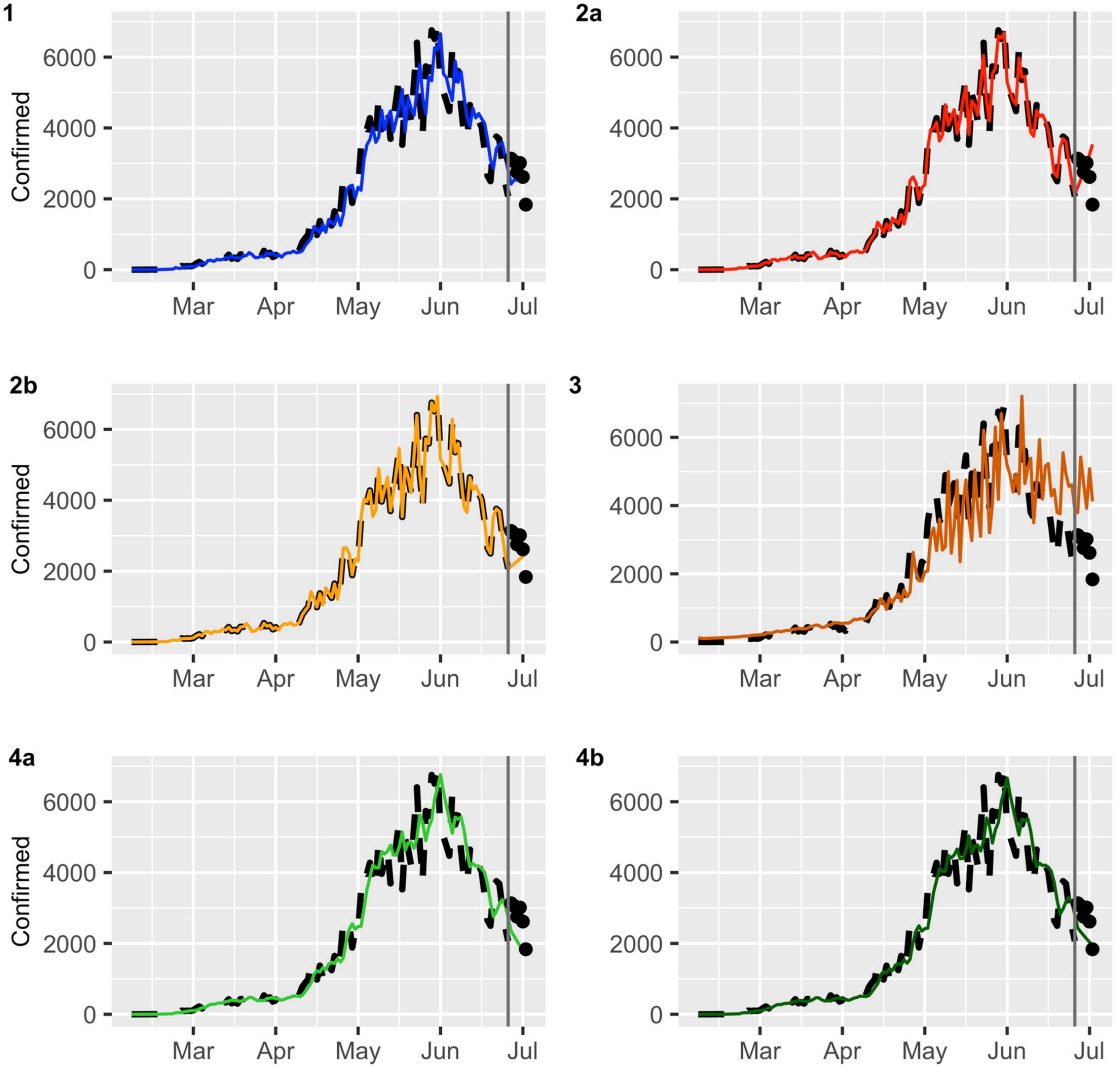
Multistep forecasts for confirmed COVID-19 cases (black dashed lines). The continuous line and dots represent fitted values and the ex post forecast respectively.

**Fig 7:**
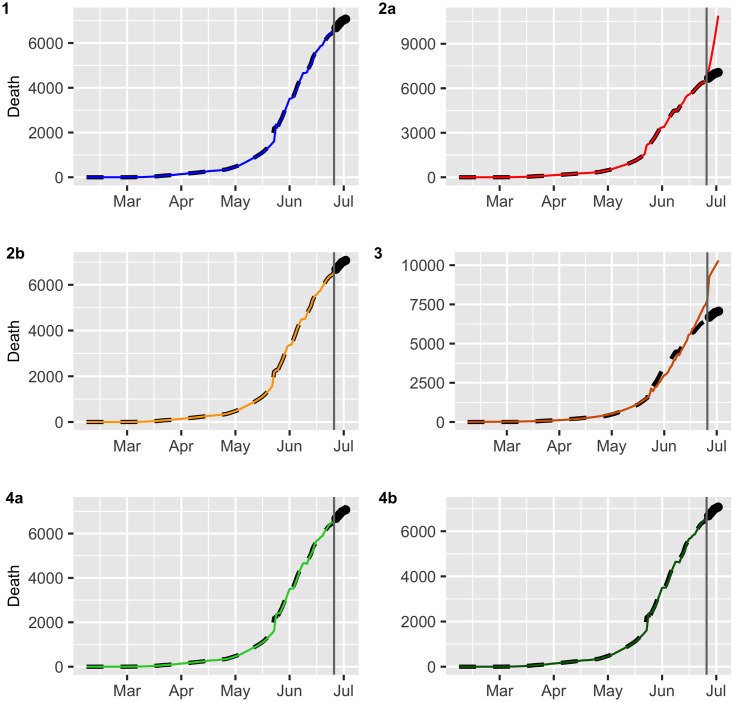
Multistep forecasts for COVID-19 deaths (black dashed lines). The continuous line and dots represent fitted values and the ex post forecast respectively.

In this section, we evaluate the accuracy of the predictions by using a training set and test set. We consider a training set {*X*_*t*_} from March 02 to July 06 (estimation period) with a total of *N* = 129 observations and test data {*X*_*t*_} from July 07 to July 14 (validation period) which will be used for *m*-step-ahead prediction with *m* = 6 daily observations. The values ${\hat{X}}_{N+1},\ldots ,{\hat{X}}_{N+m}$ are called the ex post forecast or period forecast with the starting period on July 07. The *m*-step-ahead forecasts are compared with the validation period, giving rise to ex post forecast errors, i.e., $X_{N+h}-{\hat{X}}_{N+h}$ for horizon *h* = 1, …, *m*. The errors were assessed by the statistics of the residuals, such as the mean error (ME), RMSE, mean absolute error (MAE), mean percentage error (MPE) and mean absolute percentage error (MAPE), where small values of these statistics reflect a goodness of fit criterion of the model used. Table 3 reports the statistics of ex post forecast errors of the models on both datasets. In the case of confirmed data, all indicators favor the ARIMA model, i.e., Model 1 with an MAPE of the 17.5%, and for the death data, all the statistics favor the damped trend method, i.e., Model 4b with an MAPE equal to 0.37%. These values are reasonably low values demonstrating the suitability of the proposed model for prediction. The above findings are supported by Figs 6 and 7.

**Table 3:** The descriptive statistics of ex post forecast errors.

Dataset / Model	ME	RMSE	MAE	MPE	MAPE
**Confirmed** Model 1	-222.3229	510.1141	454.268	4.903861	17.53703
Model 2a	-164.3078	838.1199	614.4429	-12.22922	26.74773
Model 2b	422.8959	693.6704	643.8252	11.81886	23.85205
Model 3	-1764.664	1904.865	1764.664	-69.39226	69.39226
Model 4a	735.086	784.1449	735.086	25.87431	25.87431
Model 4b	551.5119	635.8757	587.9894	18.47044	20.45723
**Death** Model 1	-56.8675	75.45366	56.8675	-0.81314	0.81314
Model 2a	-2018.527	2320.163	2018.527	-28.93521	28.93521
Model 2b	138.3735	145.9142	138.3735	1.992866	1.992866
Model 3	-2891.794	2900.363	2891.794	-41.84812	41.84812
Model 4a	-83.45833	109.3747	83.45833	-1.191752	1.191752
Model 4b	-16.01247	39.42867	26.08101	-0.22479	0.371870

## 4 Conclusions

In this paper, we have proposed a comparative analysis of the most widely used time series models in sequential data modeling. In particular, an ARIMA model, a State Space model, a Bayesian model for counting data, and Exponential Smoothing techniques have been proposed. The main motivation of this study is to contribute to the discussion on the types of mathematical models that can be used for making predictions of the number of confirmed COVID-19 diagnoses and the number of deaths from COVID-19 and thus provide relevant information for timely decision-making by the Chilean government.

In regard to Chile’s COVID-19 dataset and based on the class of models considered in this study, we can say that for cases with a confirmed diagnosis of COVID-19, the best model corresponds to the well-known ARIMA model, whereas for cases of deaths from COVID-19 in Chile, the best model resulted in Damped Trend method. In line with other authors, we can affirm that the proposal of this study does not imply its global use in the prediction of confirmed cases and deaths from COVID-19, since the performance of this model is subject to the biopsychosocial determinants of each country. A possible generalization of our study is to develop machine learning techniques to model the behavior of these curves, subject to the availability of large volumes of data. Furthermore, a statistical analysis can be of merit to find relationships between the spread of the virus and biopsychosocial determinants of Chilean health.
